# Transcatheter Aortic Valve Implantation in a Nonagenarian with Aortic Aneurysm: Futility or Utility?

**DOI:** 10.1155/2018/5434953

**Published:** 2018-02-01

**Authors:** Evelyn Fennelly, Marcus Lee, Mark Da Costa, Sherif Sultan, Faisal Sharif, Darren Mylotte

**Affiliations:** ^1^Department of Cardiology, University Hospital Galway, Galway, Ireland; ^2^Department of Cardiothoracic Surgery, University Hospital Galway, Galway, Ireland; ^3^Department of Vascular Surgery, University Hospital Galway, Galway, Ireland

## Abstract

Transcatheter aortic valve implantation (TAVI) has emerged as the standard of care for older patients with symptomatic severe aortic stenosis (AS) at high or excessive operative risk. There remain patients that are of such considerable risk that even TAVI can be futile. Such patients present ethical conundrums for institutional heart teams. Herein we present a case of a 90-year-old female patient with symptomatic severe AS and significant comorbidities including diffuse peripheral vascular disease and a large ascending aortic aneurysm. Would TAVI be utile or futile in this patient?

## 1. Introduction

While transcatheter aortic valve implantation (TAVI) is now the standard of care for elderly patients with symptomatic severe aortic stenosis (AS) at high or excessive operative risk, there remain, however, patients of such high risk that even TAVI may be futile. Such cases present the institutional Heart Team with many difficult considerations and ethical dilemmas. We illustrate these issues through the case presentation of a nonagenarian patient with severe symptomatic AS, multiple comorbid illnesses, and anatomical contraindications to TAVI.

## 2. Case Presentation

We present the case of a 90-year-old female with symptomatic severe AS. She described an episode of presyncope and progressive dyspnea on exertion (NYHA Class III). Medical history included hypertension, hypercholesterolemia, paroxysmal atrial fibrillation, stroke, peripheral vascular disease (PVD), and an ascending aortic aneurysm. She lived independently with Katz index of 6/6. Transthoracic echocardiogram (TTE) confirmed severe AS (mean gradient 40 mmHg; aortic valve area 0.8 cm^2^), concomitant moderate aortic regurgitation, and preserved left ventricular function (ejection fraction 55%).

**Figure 1:**
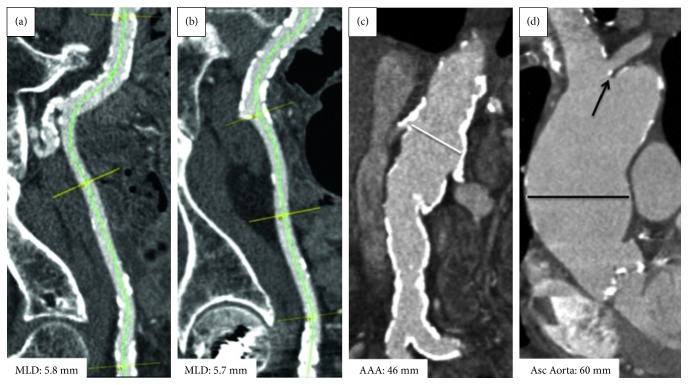
(a) MSCT of the right illiofemoral artery demonstrating an MLD of 5.8 mm. (b) MSCT of the left illiofemoral artery demonstrating heavy calcification just at the level of the femoral head and an MLD of 5.7 mm. (c) MSCT of the descending aorta. The white line denotes the maximum diameter of 46 mm. (d) MSCT of the ascending aorta. The black line denotes the maximum diameter of the ascending aorta aneurysm measuring 60 mm. The black arrow shows the left subclavian entering the right brachiocephalic trunk (the bovine arch).

**Figure 2:**
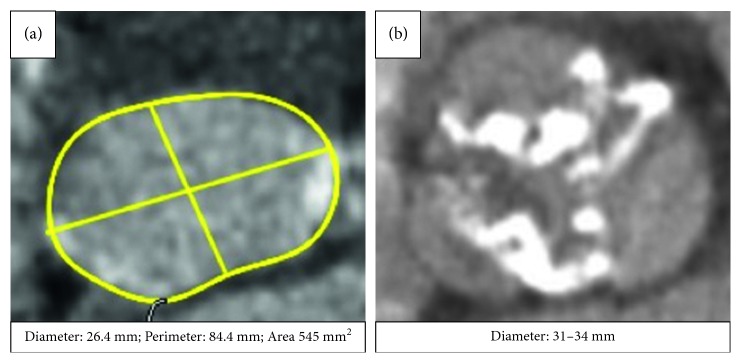
(a) MSCT of the aortic annulus with the diameter of 26.4 mm, perimeter of 84.4 mm, and aortic annular area of 542 mm^2^. (b) MSCT of the aortic valve demonstrating a tricuspid aortic valve with heavy calcification of the aortic valve leaflets.

Given the patient's severe symptoms, advanced age, and associated comorbid illnesses, the patient was considered for TAVI. Multislice computed tomography (MSCT) showed a heavily calcified, tortuous iliofemoral vasculature (minimal lumen diameter (MLD) right: 5.7 mm; left: 5.8 mm), small subclavian arteries (MLD: 4.5 mm), and a bovine aortic arch (Figure 1). MSCT identified an infrarenal abdominal aortic aneurysm (AAA) (maximum diameter: 46 mm). The diameter of the ascending aortic aneurysm was 60 mm. The trileaflet aortic valve was heavily calcified (annular dimensions: mean diameter 26.4 mm; area 542 mm^2^; perimeter 84.4 mm) (Figure 2).

The institutional Heart Team considered her to be at excessive risk for surgical aortic valve replacement (SAVR): EuroSCORE II 17.10%; Society of Thoracic Surgeons predicted risk of mortality 7.9%. A conservative management strategy was initially considered; however the patient and her family pressed for TAVI due to debilitating symptoms. Given the ascending aorta and aortic branch anatomy, a transfemoral TAVI with a surgical femoral arterial cut-down was considered to be the most appropriate vascular access route for implantation of a self-expanding transcatheter heart valve (THV). The periprocedural plan did not include transition to cardiopulmonary bypass as the patient had been declined traditional cardiac surgery by the institutional Heart Team.

Under general anesthesia, the left common femoral artery was exposed, and an 18 Fr Cook (Cook Inc., Bloomington, IN, USA) vascular access sheath was inserted just cranial to a heavily calcified arterial segment. Sheath advancement to the descending aorta was challenging, though ultimately successful (Video 1). Heparin was administered to maintain an activated clotting time of >300 seconds. The stenotic aortic valve was crossed with some difficulty, and a stiff guide wire was placed in the left ventricle. Preimplant balloon aortic valvuloplasty was performed with an 18 mm NuMED balloon (NuMED Inc., Hopkinton, NY, USA) under rapid ventricular pacing. A 31 mm Medtronic CoreValve (Medtronic, Minneapolis, MN, USA) was deployed at an appropriate depth (3–6 mm), and transesophageal echocardiography and aortography suggested excellent valve function with mild paravalvular aortic regurgitation (Videos 2 and 3).

**Figure 3:**
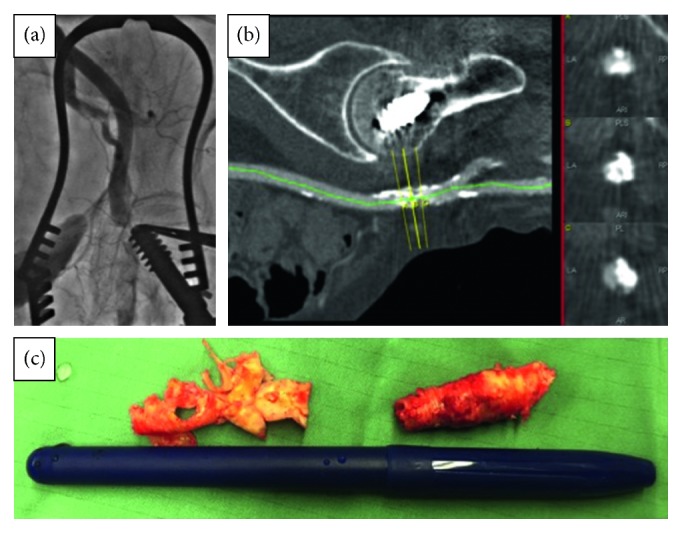
(a) Control angiography showing complete occlusion of the left femoral artery just distal to the puncture site. (b) MSCT of the left femoral artery showing heavy calcification at the level of the femoral head. Red arrow shows puncture site. (c) Calcific atheroma from the left common femoral artery during the femoral endarterectomy.

Closure of the femoral arteriotomy was performed with a 6.0 Prolene. Control femoral angiography, however, showed occlusion of the common femoral artery just caudal to the puncture site (Figure 3(a); Video 4), and an area of extensive calcific plaque was observed on the screening MSCT (Figure 3(b)). In light of the extensive calcific disease, it was deemed that percutaneous recanalization of the artery would be impossible, and a common femoral endarterectomy was performed (Figure 3(c)). The patient subsequently required further surgical exploration for ongoing bleeding and needed transfusion with 10 units of packed red blood cells. She recovered quickly and was ambulating independently on discharge to respite care (postoperative day 8).

**Figure 4:**
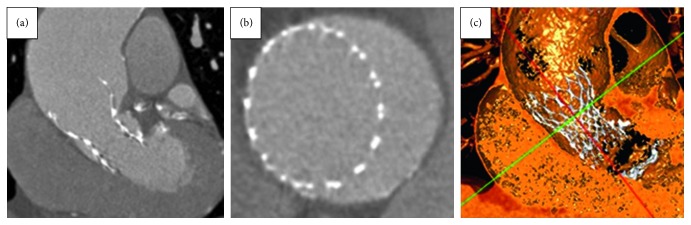
(a) MSCT of the 31 mm CoreValve in the aorta postimplant. (b) Aortic end of the CoreValve “floating” in the aorta. (c) 3D reconstruction of the CoreValve showing apposition to the greater curvature of the aorta.

The patient underwent routine follow-up, including outpatient review and surveillance echocardiography. Follow-up TTE at 6 months showed normal valve function, mean gradient of 6 mmHg, and mild paravalvular leak. Repeat MSCT showed a well-positioned valve, with the outflow floating in the ascending aortic aneurysm, without evidence of in situ thrombus, and no expansion of the ascending or descending aortic aneurysms (Figure 4). The patient remains asymptomatic at 12-month follow-up and will continue to undergo surveillance echocardiography annually.

## 3. Discussion

We present a challenging case of a nonagenarian with severe symptomatic AS, multiple comorbid illnesses, and anatomical contraindications to TAVI. This case presented an ethical dilemma to the Heart Team, given the extreme patient risk. Initially, we preferred a conservative management strategy, but when faced with the patient's preference for definitive therapy, we elected to undertake transfemoral TAVI.

This case underscores the importance of a multidisciplinary approach to TAVI. The successful outcome for our patient was achieved with the collaboration of the cardiology, cardiothoracic, and vascular surgery teams, anesthesia, elderly medicine, and support services. The Heart Team model of care is recommended in both European and U.S. guidelines, which cite its role as essential in optimizing patient care [1, 2].

Vascular access was particularly challenging in this case; the extensive severe PVD left few options for insertion of an 18 Fr vascular access sheath. Percutaneous transfemoral, transaortic, and transcarotid approaches were ruled out due to the calcific nature and small caliber of the femoral arteries, the aneurysmal ascending aorta, and the bovine aortic arch, respectively [3]. Transapical TAVI could have been an option, though may be associated with inferior clinical outcomes [4]. Transcaval TAVI has also emerged as a possible access route for no-option patients, though experience is limited [5]. Ultimately, we elected to push the boundaries of the transfemoral approach using a surgical cut-down to try minimize patient risk. The left common femoral artery MLD of 5.7 mm yielded a sheath to femoral artery ratio (SFAR) of 0.95. In calcified vessels, a SFAR > 1.0 is recommended to avoid vascular complications [6]. When the complication was encountered, the vascular team was on hand to undertake emergent surgery.

Another major challenge in this case was the ascending aortic aneurysm. Aortic root dilation occurs in up to 3% of patients with severe AS [7]. Current American College of Cardiology recommendations suggest concomitant aneurysm repair during SAVR in those with an ascending aorta or aortic root > 45 mm in diameter [8]. Such recommendations do not apply to inoperable patients; the role of TAVI here remains unclear. Aortic root replacement aims to prevent future catastrophic dissection or rupture by restoring the normal dimension of the ascending aorta with a graft [9]. Untreated, such aneurysms remain exposed to the high velocity jet created by the stenotic valve, and this wall stress may induce dissection or rupture. The annual risk of death, rupture, or dissection for ascending aortic aneurysms of 60 to 69 mm in diameter is estimated to be 15.6% per year [10]. Critically, the risk of rupture increases 27-fold after 60 mm as there is an annual mean growth rate of 1 mm per year [10]. One could hypothesize that replacing the aortic valve alone (TAVI in this case) could reduce wall stress by removing the high-velocity jet caused by the stenotic valve [11, 12]. In contrast, the intervention itself could destabilize the aneurysm, and indeed the self-expanding prosthesis itself could exert forces on the aortic wall [9]. There are currently no published data on long-term changes in ascending aortic aneurysm sizes after TAVI.

Valve thrombosis has recently been identified as a complication of bioprosthetic valve replacement, occurring in up to 40% of recipients [13]. This entity may be prosthesis-dependent and may be associated with atrial fibrillation [14]. An ascending aortic aneurysm may increase the risk of valve thrombosis, according to the principles of Virchow's triad [15]. In this case, there was no evidence of valve thrombosis on MSCT after 6-month treatment with dual antiplatelet therapy.

The case presented here demonstrates the importance of the institutional Heart Team in optimizing patient outcome with TAVI. We suggest cautiously that CoreValve implantation for inoperable older patients with ascending aortic root aneurysms is feasible and safe and in our patient was associated with symptomatic improvement.

## Supplementary Materials

Supplementary Materials Video 1: challenging—though ultimately successful—sheath advancement to the descending aorta. Video 2: aortography showing a 31 mm Medtronic CoreValve (Medtronic, Minneapolis, MN, USA) deployed at an appropriate depth. Video 3: transoesophageal echocardiography showing excellent valve function with mild paravalvular aortic regurgitation in the deployed 31 mm Medtronic CoreValve (Medtronic, Minneapolis, MN, USA). Video 4: control femoral angiography showing occlusion of the common femoral artery just caudal to the puncture site.
